# Animal versus plant protein and adult bone health: A systematic review and meta-analysis from the National Osteoporosis Foundation

**DOI:** 10.1371/journal.pone.0192459

**Published:** 2018-02-23

**Authors:** Marissa M. Shams-White, Mei Chung, Zhuxuan Fu, Karl L. Insogna, Micaela C. Karlsen, Meryl S. LeBoff, Sue A. Shapses, Joachim Sackey, Jian Shi, Taylor C. Wallace, Connie M. Weaver

**Affiliations:** 1 Department of Public Health and Community Medicine, School of Medicine, Tufts University, Boston, MA United States of America; 2 Friedman School of Nutrition Science and Policy, Tufts University, Boston, MA United States of America; 3 Yale Bone Center at the Yale School of Medicine, Yale University, New Haven, CT United States of America; 4 Skeletal Health and Osteoporosis Center and Bone Density Unit; Harvard Medical School, Boston, MA United States of America; 5 Endocrine, Diabetes and Hypertension Division, Brigham and Women's Hospital, Boston, MA United States of America; 6 Department of Nutritional Sciences, Rutgers University, New Brunswick, NJ United States of America; 7 Department of Nutritional Sciences, Rutgers School of Health Professions, Newark, NJ United States of America; 8 Department of Nutrition and Food Studies, George Mason University, Fairfax, VA United States of America; 9 Think Healthy Group, Inc, Washington DC United States of America; 10 Department of Nutrition Science, Women’s Global Health Institute, Purdue University, Nutrition Science, West Lafayette, IN United States of America; University of Arkansas for Medical Sciences College of Pharmacy, UNITED STATES

## Abstract

**Background:**

Protein may have both beneficial and detrimental effects on bone health depending on a variety of factors, including protein source.

**Objective:**

The aim was to conduct a systematic review and meta-analysis evaluating the effects of animal versus plant protein intake on bone mineral density (BMD), bone mineral content (BMC) and select bone biomarkers in healthy adults.

**Methods:**

Searches across five databases were conducted through 10/31/16 for randomized controlled trials (RCTs) and prospective cohort studies in healthy adults that examined the effects of animal versus plant protein intake on 1) total body (TB), total hip (TH), lumbar spine (LS) or femoral neck (FN) BMD or TB BMC for at least one year, or 2) select bone formation and resorption biomarkers for at least six months. Strength of evidence (SOE) was assessed and random effect meta-analyses were performed.

**Results:**

Seven RCTs examining animal vs. isoflavone-rich soy (Soy+) protein intake in 633 healthy peri-menopausal (n = 1) and post-menopausal (n = 6) women were included. Overall risk of bias was medium. Limited SOE suggests no significant difference between Soy+ vs. animal protein on LS, TH, FN and TB BMD, TB BMC, and bone turnover markers BSAP and NTX. Meta-analysis results showed on average, the differences between Soy+ and animal protein groups were close to zero and not significant for BMD outcomes (LS: n = 4, pooled net % change: 0.24%, 95% CI: -0.80%, 1.28%; TB: n = 3, -0.24%, 95% CI: -0.81%, 0.33%; FN: n = 3, 0.13%, 95% CI: -0.94%, 1.21%). All meta-analyses had no statistical heterogeneity.

**Conclusions:**

These results do not support soy protein consumption as more advantageous than animal protein, or vice versa. Future studies are needed examining the effects of different protein sources in different populations on BMD, BMC, and fracture.

## Trial registration

**Clinical trial registry number and website:** PROSPERO registry #: **CRD**42015017751 http://www.crd.york.ac.uk/PROSPERO/display_record.asp?ID=CRD42015017751.

## Introduction

Bone undergoes continuous remodeling; therefore, adequate supply of amino acid and mineral substrate are needed to support the formation of new bone. Protein has been identified as being both beneficial and detrimental to bone health depending on a variety of factors including the type of protein in the diet, the protein source, calcium intake, the population (i.e., older, gender), weight loss, and/or the acid-base balance of the diet [1]. Dietary protein has long been known to increase renal calcium excretion [2]. This increase in urinary calcium is a result of buffering the metabolic acid load of dietary sulfur-containing amino acids largely present in animal-derived proteins by releasing alkaline stores (e.g., calcium salts) from bone. More recently, it has been shown with dual stable calcium isotope and balance studies that the increase in urinary calcium can be accounted for by improved absorption efficiency with no change in calcium balance [3–5]. If the lack of difference in calcium absorption and balance with protein intake in short term studies is assumed to persist long term, there may be no difference in bone mineral density (BMD) due to dietary protein level or type.

In our recent systematic review, commissioned by the National Osteoporosis Foundation (NOF), we reported the effect of level of dietary protein on bone. We concluded that low to moderate evidence suggests no adverse effects of higher compared with lower total protein intake on bone BMD outcomes. Only the lumbar spine BMD had moderate evidence supporting benefits of higher protein intake. There was moderate evidence that there was no relation between higher vs lower protein intake and hip fractures, but inadequate evidence for all other long-term fracture or fall outcomes [6].

Here we report the effect of source of protein (animal vs. plant) on bone health. Intakes of several amino acids, including alanine and glycine, have been associated with higher BMD independent of an individual’s genetic background [7]. In particular, aromatic amino acids found at higher levels in animal-derived proteins have been suggested to increase calcium absorption through their binding to the calcium sensing receptor (CaR) [8]. However, the effects of animal versus plant proteins that contain dissimilar amino acid profiles remains a subject of great debate; some plant proteins have the potential of producing more mEq of sulfuric acid per g of protein than some animal proteins (e.g., wheat vs. beef). Therefore, the NOF commissioned an extended systematic review of the scientific literature on this research question so that clear evidence-based public health recommendations may be developed for consumers. However, due to the paucity of studies examining this research question, it is important to note that the included intervention studies focus only on soy protein as the plant protein source of interest.

## Methods

The PRISMA (Preferred Reporting Items for Systematic Reviews and Meta-analyses) statement was followed in reporting this systematic review [9]. A prospectively developed protocol for this systematic review was registered on PROSPERO [10]. The materials and methods are described in a previous paper [6]. Search strategies were developed in consultation with two librarians. The initial strategy was developed for Ovid Medline® (1946 to week 4 October 2016) and then adjusted for four additional electronic databases: Cochrane Central Register of Controlled Trials (1991 to 31 October 2016), Scopus (+ EMBASE 1974 to 31 October 2016), Web-of-Science (1864 to 31 October 2016) and Global Health (1910 to 31 October 2016). All searches were limited to the English language and human studies that examined the relationships of dietary protein intake (via foods or supplements) on bone health outcomes of interest. The complete search strategy is presented in **S1 Table**).

### Study eligibility criteria

We included intervention trials that compared equal amounts of dietary protein from different sources (animal vs. plant) and prospective cohort studies that examined different doses of dietary protein intake (i.e., animal and plant protein intake) and their effects on bone health outcomes of interest in healthy adults ≥ 18 years of age. We excluded studies comparing varying doses of protein intake from the same source (i.e., high vs. low total protein intake, covered in a previous review [6]). Healthy adults were defined as “healthy obese” adults; those with past or current fractures but no other pre-existing conditions; older adults with sarcopenia or frailty but no other clear disease (e.g., renal disease); and adults ≥45 years of age with hypertension, due to its higher prevalence among older adults [11]. We excluded studies among children and adolescents (i.e., less than 18 years of age), pregnant or lactating women, participants who were all diagnosed with a particular disease (e.g., 100% of participants with type 2 diabetes), and studies where > 20% of the baseline population had a disease. Outcomes of interest included: BMD, total body bone mineral content (BMC), and bone fracture or fracture risk of any site in studies ≥ one year in duration, as defined in previous studies [12]; falls; and biomarkers for bone metabolism, mineralization, formation, turnover or resorption if the study was ≥ six months in duration. As studies among those <18 years of age were excluded, we excluded bone development outcomes. The complete list of bone health outcomes is described in **Table 1**.

**Table 1:** Included bone outcomes of interest^1^^,^^2^.

◦ Bone mineral content (BMC): total body (TB) only ◦ Bone mineral density (BMD): TB, total hip (TH), femoral neck (FN), lumbar spine (LS) ◦ Fracture: all sites ◦ Falls ◦ Bone quality ◦ Bone metabolism biomarkers^2^ bone-specific alkaline phosphatase (BSAP), collagen type 1 cross-linked C-terminal telopeptide (CTX), collagen type 1 cross-linked N-telopeptide (NTX), deoxypyridinoline (DPD)

^1^ The following outcomes were included outcomes of interest, but were not reported in the included studies: bone quality (e.g., via amplitude-dependent speed of sound (Ad-SOS), bone sialoprotein (BSP), tartrate-resistant acid phosphatase 5b, osteocalcin (OC), alkaline phosphatase (ALP), C- or N-terminal type 1 procollagen (C1NP or P1NP), hydroxyproline, pyridinoline (PYD)

^2^ Ratios of the biomarkers (e.g., with creatinine) were included as outcomes of interest

### Study selection process

Citations identified from the non-Medline databases were first title-screened by a single investigator to exclude in vitro, cell and stem cell studies, animal studies, cross-sectional studies, retrospective case-control studies, interrupted time series studies, meta-analyses, and review articles. Next, all abstracts identified in the literature searches were independently screened by two or more investigators using Abstrackr™ [13]. Full-text articles were retrieved for all potentially relevant abstracts and independently screened by two investigators based on eligibility criteria. In any instance where studies were conducted in the same cohort population and time period, the first published study was retained. All screening conflicts were resolved via consensus by the entire research team.

### Data extraction

The data extraction forms used in a previous systematic review evidence report [12] were modified for our topic of interest. The following items were extracted: study characteristics, baseline population characteristics, background diet data, dietary assessment methods, interventions (for intervention studies only), confounders and effect modifiers used in statistical analysis, relevant outcomes assessed, and results (complete data extraction forms are available upon request). Results were extracted quantitatively when reported and, when absent, qualitatively results were extracted. For all studies, in order of preference, multivariate adjusted analyses were extracted over age adjusted or crude measures and risk and hazard ratios over incidence ratios and odds ratios. Each study was extracted by one investigator and reviewed and confirmed by a second investigator. Any disagreements were resolved via group consensus.

### Risk of bias (ROB) in individual studies

Intervention studies’ methodological quality were assessed using a modified version of the Cochrane risk of bias (ROB) tool [14]. This tool was modified for nutrition and outcome-specific items and addressed risk of selection bias, performance bias, detection bias, attrition bias, reporting bias, and other potential biases. Two investigators independently conducted ROB assessments and any disagreements resolved via group consensus. It is important to note that certain categories were given greater weight when deciding on overall ratings. For example, an unclear rating for blinding of outcome assessors was given less weight for outcomes with objective measurements (e.g., fractures confirmed by x-ray or BMD outcomes measured clinically).

### Data synthesis

All included studies were summarized in narrative form and in summary tables that tabulated key features of the study populations, design, intervention, outcomes, and results. Summary tables were organized by study type (i.e., randomized control trial (RCT) vs. cohort study) and study results were qualitatively and quantitatively summarized first by outcome of interest, then by group comparison (isoflavone-rich soy protein vs. animal protein and, where applicable, isoflavone-poor soy protein vs. animal protein and isoflavone-rich soy protein vs. isoflavone-soy protein).

#### Qualitative synthesis

The strength of evidence (SOE) for major comparisons and outcomes was assessed through a consensus process of the entire research team, using a modified version of the NOFs evidence grading system [15] and the grading system utilized by the American Diabetes Association and other prominent groups [15–17]. As detailed previously [6], SOE levels used were A (Strong), B (Moderate), C (Limited), D (Inadequate), E (Expert Consensus or Clinical Experience), or NA (not applicable).

#### Meta-analysis (quantitative synthesis)

The methods outlined in the Cochrane Handbook for conducting meta-analysis of RCTs were followed [18].

For eligible BMD outcomes in RCTs, we used the reported or calculated net percentage change between the animal and plant protein groups as the effect size measure in the meta-analysis because most of RCTs reported within-group percentage changes in BMD outcomes. The mean within-group percentage change, if not reported, was calculated using post-intervention mean minus baseline mean, then divided by baseline mean and multiplied by 100%. Its standard deviation (SD) was estimated using the SD of the mean change divided by the baseline mean. The net percentage change was the difference in the within-group percentage changes of the two groups using the animal protein group as the reference group. Thus, a positive net percentage change indicates an effect (i.e., less bone loss) favoring plant protein intake. The SD of the net percentage change was the pooled SD of the two SDs of the mean change: SD_pooled_ = √(((n1−1) x SD_1_ + (n2−1) x SD_2_) / (n1 + n2−2)) where n1 and n2 are the sample sizes, and SD_1_ and SD_2_ are the SDs of the mean within-group percentage change of the plant and animal protein groups, respectively. For the one study [19] that did not report final sample sizes, the baseline sample size was used in our calculations. We assumed a correlation coefficient (Corr) value of 0.50 to impute the missing SD of the mean within-group change. Sensitivity analyses using Corr values of 0.20 and 0.80 were conducted to evaluate the impacts of the correlation assumptions on the meta-analysis results, and none showed appreciable impacts **(S2 Table)**.

In light of clinical heterogeneity (different doses of protein interventions), we performed random-effects meta-analyses for outcomes with at least three unique RCTs [20]. We used both the Q statistic (considered significant when the *P* value was less than 0.10) and the *I*^*2*^ index to quantify the extent of statistical heterogeneity [18]. We defined low, moderate and high heterogeneity as *I*^*2*^ values of 25%, 50% and 75%, respectively. These cutoffs are arbitrary and were used for descriptive purposes only [21]. Studies were excluded from meta-analyses if required information for the aforementioned calculations were not reported for any given outcome. One original author was contacted to obtain missing quantitative data needed for meta-analysis, but the data were no longer available [22].

All calculations and meta-analyses were conducted in Stata SE 13 (Stata Corp). The analytical datasets can be found in the **S1 File**. Two-tailed P values less than 0.05 were considered statistically significant.

## Results

**Fig 1:**
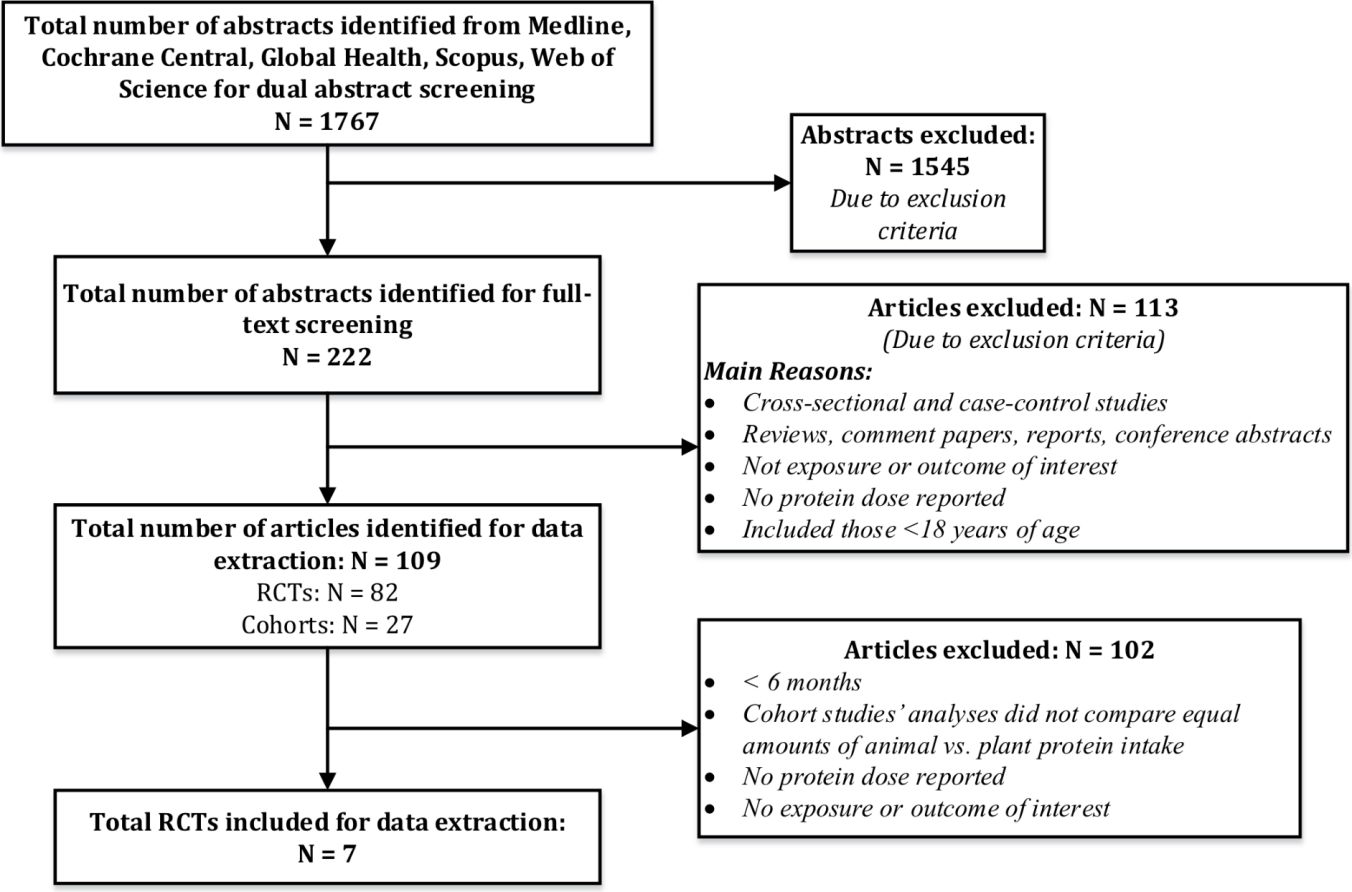
Literature search and study selection process.

Our search yielded 1,767 citations for dual abstract screening, of which 222 were identified for full-text screening and seven RCTs were finally included. No cohort studies met the eligibility criteria. Details of the literature search and study selection flow are summarized in **Fig 1**. Ten prospective cohort studies were excluded because there were no analyses directly comparing animal to plant protein intake in relation to the outcomes of interest. However, the results from these cohort studies are briefly described in the end of result section to summarize the associations between varying intakes of plant or animal protein and long-term bone health outcomes as supplementary information to the findings from RCTs.

### Characteristics of RCTs

Seven RCTs [19, 22–27] that met the eligibility criteria all compared soy protein to animal protein intake in generally healthy peri- or postmenopausal women (**Table 2**). A total of 803 and 633 women were enrolled in the studies and analyzed, respectively. Though all plant protein sources administered in these studies were soy protein, animal protein sources were not uniform across studies: five [19, 22, 25–27] administered milk protein (whey with or without casein protein), one [23] administered a mix of milk and egg white protein, and one [24] administered an unspecified, non-soy protein. Furthermore, protein doses administered varied across studies: one [23] gave participants 18 g protein/d, four [19, 24, 25, 27] gave 25–25.6 g protein/d, and two supplemented with higher doses of 38 g protein/d [26] and 40 g protein/d [22]. Calcium supplement doses also varied by study. One study [23] only supplemented 315 mg calcium/d to participants unable to achieve dietary intakes of 1200–1500 mg calcium/d. A second study [25] did not specify the calcium dosage supplemented to all participants. All participants in the remaining five studies [19, 22, 24, 26, 27] received calcium supplementation ranging from 500–1200 mg/d. The intervention durations ranged from 6 to 24 months.

**Table 2:** Study characteristics summary table, RCTs (N = 7)^1^.

First author, pub year [ref] (country); study design	Participants	Total enrolled/ analyzed (N) [F%]	Baseline mean age (SD)	BMI (kg/m^2^) or body weight (kg), Mean (SD)	Group names	Sources of dietary interventions	Interventions (protein total daily dose)	Co-Interventions	BL Dietary Protein intake (g/d) Mean (SD)	BL Dietary Ca intake (mg/d) Mean (SD)	Study Duration (mo)	Outcomes assessed (Endpoint (1°, 2° or NR))	Funding Source
Alekel 2000 [22] (USA) parallel RCT	Healthy, peri-menopausal	80/69 [100]	Median 50.6 (NR)	BMI: SPI-: 24.2 (3.1) SPI+: 24.0 (3.6) Control: 23.9 (3.0)	Isoflavone-poor soy protein (SPI-), isoflavone-rich soy protein (SPI+), whey protein (Control)	Food and protein powder- mix w/ food or beverage	SPI groups: 40g soy protein Control: 40g whey protein	SPI+: 80.4 mg/d aglycone components/d SPI-: 4.4 mg aglycone components/d All: 650 mg Ca/d	NR	SPI-: 883(349) SPI+: 790(331) Whey: 761(285)	6	BSAP, NTX (2°)	combo (gov, ind)
Arjmandi 2005 [24] (USA) parallel RCT	Healthy post-menopausal	87/62 [100]	Crude NR Soy: LSM: 56 (SE: 5) Control: LSM: 53 (SE: 6)	BMI: SPI+: LSM: 28.6 (SE 0.9) Control: LSM: 27.3 (SE 1.0)	Soy protein (SPI+), Control	Food and drink mix	SPI+: 25g soy protein Control: 25g non-soy protein	SPI+: 60 mg isoflavones/d All: 500 mg Ca/d	SPI+: LSM: 75.8 (SE 3.6) Milk: LSM: 64.2 (SE 4.1)^1^	SPI+: LSM: 873 (SE:60) Milk: LSM: 796 (SE:69)	12	BMD (TB, TH, LS), TB BMC, DPD, BSAP (1°)	combo (gov, ind)
Evans 2007 [19] (USA) parallel RCT	Healthy, post-menopausal	61/43 [100]	62 (5)	Weight: SPI+: 67.6 (7.3), SPI+Ex: 66.7 (13.3) MPI: 73.0 (11.4) MPI+Ex: 68.7 (14.1)	Soy protein isolate (SPI+), SPI+exercise (SPI+Ex), milk protein isolate (MPI), MPI +exercise (MPI+Ex)	Beverage	SPI groups: 25.6g soy protein MPI groups: 25.6g milk protein	SPI groups only: 91.2mg isoflavones/d All: 900 mg Ca/d and 125 IU Vit D/d	No sig diff between groups at BL, data NR	No sig diff between groups at BL, data by group NR; All: 1,582 (382)	9	BSAP, CTX (1°)	Combo (gov, ind)
Kenny 2009 [23] (USA) parallel RCT	Healthy, post-menopausal	131/97 [100]	73.1 (5.9)	BMI: SPI-: 27.6 (4.3) SPI+: 29.3 (6.9) Control: 28.4 (4.8)	Soy protein + placebo (SPI-), soy protein+ isoflavones (SPI+), control protein +placebo (control), control protein + isoflavones	Powder +Tablets	SPI groups: 18g soy protein Control:18g milk and egg white protein mix	Dietary counseling every 3 mo from research dietician SPI+: 35 mg isoflavone aglycone equivalents/d All: if not achieving 1200–1500 mg/d via diet, administered 315 mg Ca/d and 200 IU Vit D/d	SPI+: 62.5 (13.7) SPI-: 65.4 (16.7) Control: 57.0 (21.9)	SPI+: 19.3 (7.7) SPI-: 19.3 (6.7) Control: 17.8 (8.1)	12	BMD (TB, FN, LS), NTX, BSAP (1°)	Gov
Kreijkamp-Kaspers 2004 [25] (Nether-lands) parallel RCT	Healthy, post-menopausal	202/175 [100]	Crude NR Soy: 66.5 (4.7) Placebo: 66.7 (4.8)	BMI: Soy: 26.5 (4.1) Placebo: 25.9 (3.5)	Soy protein + isoflavones (SPI+), milk protein (placebo)	Protein powder- mix with food or beverage	SPI+: 25.6g soy protein Placebo: 25.6g milk protein	SPI+: 99 mg isoflavones/d All: daily Ca supplement (dose NR)	Soy: 99.6 (22.4) Placebo:103.8 (22.8)	Soy: 1623 (533.8) Placebo: 1762 (583.2)	12	BMD (TH, FN, LS) (1°), BSAP (NR)	combo (gov, NP)
Murray 2003 [26] (USA) parallel RCT	Healthy, post-menopausal	39/30 [100]	Crude NR SPI+: 56.3 (7.4) Placebo: 53.0 (3.4)	BMI: 0.5mg SPI+: 24.6 (2.8) Placebo: 26.8 (5.5)	0.5 mg E2+SPI (SPI+), 1.0mg E2+SPI, 0.5 mg E2+ placebo (placebo), 1.0 mg E2+placebo	Protein powder- mix with food or beverage	SPI+: 38g soy protein isolate Placebo: 38g milk (whey and casein)	All: oral micronized E2 tablets (0.5–1.0mg); 1,200 mg Ca/d SPI+: 120 mg aglycone isoflavones/d	NR	NR	6	NTX (1°)	Ind
Vupadhya-yula 2009 [27] (USA) parallel RCT	Healthy, post-menopausal	203/157 [100]	Crude NR SPI-: 63.6 (SE 0.6) SPI+: 63.4 (SE 0.6) Milk: 63.8 (SE 0.5)	BMI: SPI-: 26.4 (SE 0.4) SPI+: 26.2 (SE 0.48) Milk: 26.0 (SE 0.4)	Soy protein without isoflavones (SPI-), Soy protein + isoflavones (SPI+), milk protein (milk)	Supplement-mix with beverage	SPI groups: 25g soy protein isolate Milk: 25g casein and whey	SPI+: included 90mg isoflavones Soy alone and Milk: NA All: 500 mg Ca/d, 125 IU/d Vit D	SPI-: 62.9 (1.9) SPI+: 64.9 (1.5) Milk: 61.3 (1.6)	SPI-: 955.7 (56.4) SPI+: 996.8 (56.7) Milk: 946.2 (53.3)	24	BMD (TB, FN, LS), NTX (1°)	Ind

^1^BL, baseline; BMC, bone mineral content; BMD, bone mineral density; BSAP, bone-specific alkaline phosphatase; Ca, calcium; CI, confidence interval; CTX, C-terminal telopeptide of type 1 collagen; diff, difference; DPD, deoxypyridinoline; E2, exogenous estradiol; F%, percent female participants; FN, femoral neck; LS, lumbar spine; LSM, least squared mean; MPI, milk protein isolate; NA, not applicable; NR, not reported; NTX, N-terminal telopeptide of type I collagen; RCT, randomized controlled trial; ref, reference; SD, standard deviation; SE, standard error; sig, significant; SPI, soy protein isolate; SPI+, isoflavone-rich soy protein isolate; SPI-, isoflavone-poor soy protein isolate; TB, total body; TH, total hip; Vit D, vitamin D.

In addition to assessing protein intervention doses, six studies [19, 22–25, 27] also examined participants’ diets via food diaries, 24-hour recalls, or food frequency questionnaires. There was no significant difference in dietary calcium intake between groups at baseline [19, 22–25, 27]. Among these, five studies reported dietary protein intake at baseline [19, 23–25, 27]. One study [27] reported that, though total protein intake was similar at baseline across groups, the milk protein group had a significantly greater increase in total protein intake (+27.9 g protein/d) post-intervention compared to the SPI- and SPI+ protein groups (+12.5 and +14.5 g, respectively). In a second study [24], there was a significant difference in total protein intake between groups at baseline but not post-intervention. There were no significant differences in total protein intake between groups at baseline nor post-intervention in the other three studies [19, 23, 25].

Four studies reported BMD as a primary outcome [19, 22–25, 27]. Five studies reported biomarkers among their primary outcomes, while one [22] reported them as secondary outcomes and the seventh study [25] did not specify. Of the seven studies, three reported power calculations [22, 25, 27]. ROB assessments for individual RCTs are described in **S3 Table**.

Results are organized first by outcome and then by group comparisons: isoflavone-rich soy vs. animal protein (i.e., our main question of interest) (N = 7); isoflavone-poor soy protein vs. animal protein (N = 3) [22, 23, 27]; and isoflavone-rich soy vs. isoflavone-poor soy protein (N = 3) [22, 23, 27]. No RCTs examined falls or fracture outcomes.

### LS BMD

Four RCTs [23–25, 27] examined isoflavone-rich soy protein vs. animal protein’s effect on LS BMD Of these, two RCTs [23, 27] had a third, isoflavone-poor soy protein group, and thus also contributed data on the comparison between isoflavone-poor soy protein to animal protein as well as on the comparison between isoflavone-poor and isoflavone-rich soy protein. Three studies administered similar protein doses of 25g/d [24, 27] and 25.6 g/d [25] in the form of a supplement powders to mix with food or beverages [25, 27] or both food and supplement powder [24], while the fourth study administered supplement powders containing 18g protein/d to mix with a food or beverage [23]. Administered doses of isoflavones in the soy protein groups ranged from 35-97mg/d, and animal protein sources varied across studies. Overall ROB was medium, due to low compliance in one study (<80%) [24] and incomplete outcome data in two studies (dropout rate >20% or no comparison between study completers and dropouts in per protocol analysis) [23, 24] (S3 Table).

**Fig 2:**
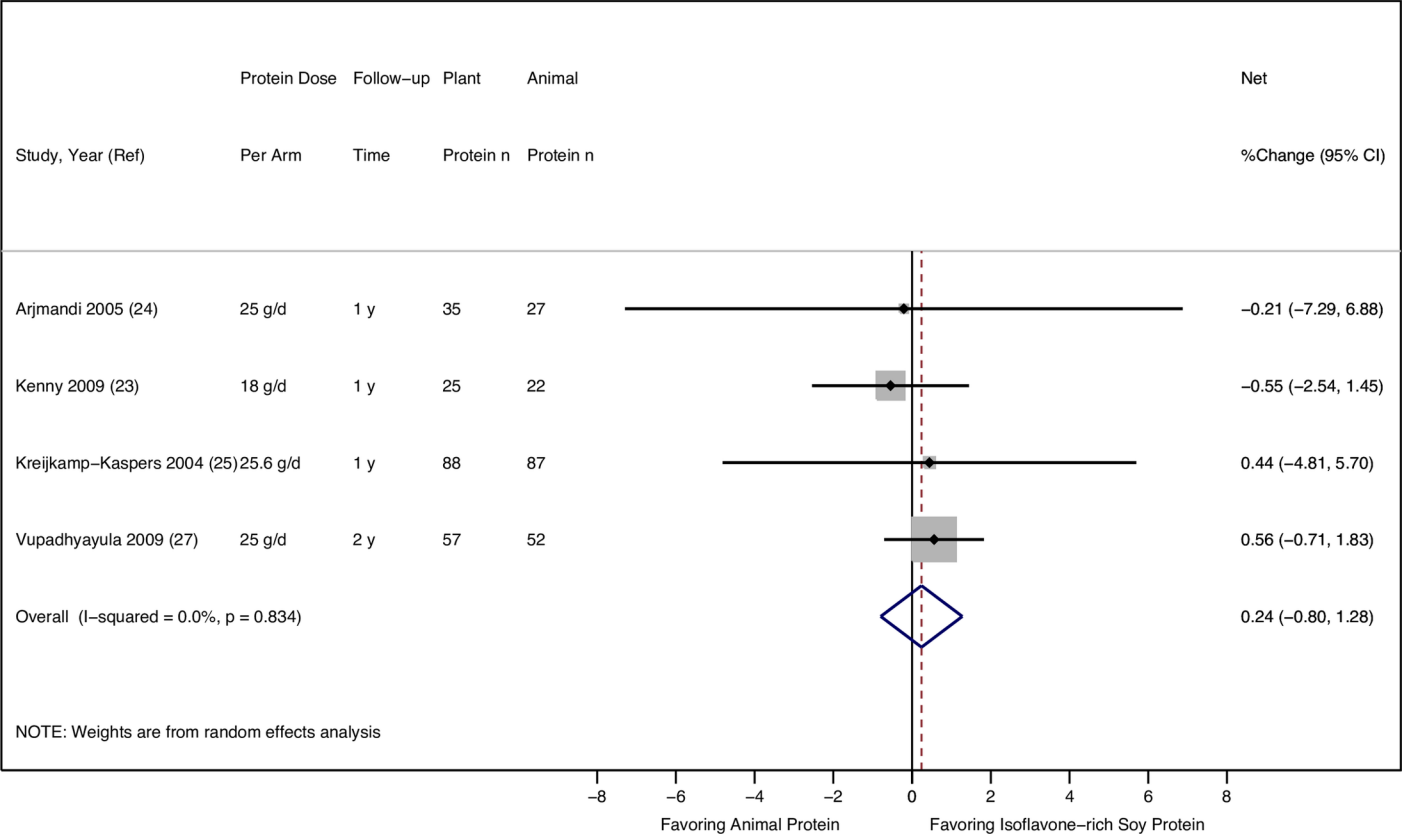
Effect of isoflavone-rich soy protein vs. animal protein intake on lumbar spine BMD in postmenopausal women.

None of the four RCTs comparing isoflavone-rich soy protein and animal protein intake found a significant difference in the net changes in LS BMD over one to two years (**Table 3**). The random-effects meta-analysis of the four RCTs in a total of 393 postmenopausal women also showed no difference in the effects of isoflavone-rich soy protein vs. animal protein intake on LS BMD, with no statistical heterogeneity (pooled mean percentage change: 0.24%, 95% CI: -0.80%, 1.28%, I^2^: 0.0%) (**Fig 2**).

**Table 3:** RCT BMD and BMC results comparing isoflavone-rich soy protein, isoflavone-poor soy protein, and animal protein^1^.

Study Year [ref]	Outcome (units)	Soy Protein (SP) (SPI+/ SPI-)	Comparator Protein (CP) (AP/ SPI-)	Study Length	SP n	CP n	SP Mean at BL	CP Mean at BL	Net change	95% CI	P-value
Kenny 2009 [23]	BMD FN (g/cm^2^)	SPI+	Milk+egg white	1 y	25	22	0.809	0.8	0.50%	-1.79%, 2.79%	>0.05
		SPI-	Milk+egg white	1 y	24	22	0.861	0.8	0.49%	-1.18%, 2.16%	>0.05
		SPI+	SPI-	1 y	25	24	0.809	0.861	0.007%	-2.24%, 2.26%	>0.05
Kreijkamp-Kaspers 2004 [25]	BMD FN (g/cm^2^)	SPI+	Milk	1 y	88	87	0.722	0.695	0.02%	-4.35%, 4.39%	0.89
Vupadhyayula 2009 [27]	BMD FN (g/cm^2^)	SPI+	Milk	2 y	57	52	0.873	0.881	0.03%	-1.23%, 1.29%	>0.05
		SPI-	Milk	2 y	48	52	0.865	0.881	-0.92%	-2.19%, 0.35%	>0.05
		SPI+	SPI-	2 y	57	48	0.873	0.865	0.95%	-2.40%, 4.30%	>0.05
Arjmandi 2005 [24]	BMD LS1-4 (g/cm^2^)	SPI+	Non-soy AP	1 y	35	27	0.944	0.941	-0.21%^2^	-7.29%, 6.88%	0.958
Kenny 2009 [23]	BMD LS2-4 (g/cm^2^)	SPI+	Milk+egg white	1 y	25	22	1.127	1.11	-0.55%	-2.54%, 1.45%	>0.05
		SPI-	Milk+egg white	1 y	24	22	1.218	1.11	-0.82%	-2.60%, 0.97%	>0.05
		SPI+	SPI-	1 y	25	24	1.127	1.218	0.27%	-1.75%, 2.30%	>0.05
Kreijkamp-Kaspers 2004 [25]	BMD LS1-4 (g/cm^2^)	SPI+	Milk	1 y	88	87	0.917	0.895	0.44%	-4.81%, 5.70%	0.79
Vupadhyayula 2009 [27]	BMD LS1-4 (g/cm^2^)	SPI+	Milk	2 y	57	52	1.085	1.104	0.56%	-0.71%, 1.83%	>0.05
		SPI-	Milk	2 y	48	52	1.076	1.104	1.17%	-0.10%, 2.44%	>0.05
		SPI+	SPI-	2 y	57	48	1.085	1.076	-0.61%	-6.00%, 4.78%	>0.05
Arjmandi 2005 [24]	BMD TH (g/cm^2^)	SPI+	Non-soy AP	1 y	35	27	0.853	0.871	-0.002%^2^	-6.28%, 6.28%	0.512
Kreijkamp-Kaspers 2004 [25]	BMD TH (g/cm^2^)	SPI+	Milk	1 y	88	87	0.861	0.831	0.49%	-3.60%, 4.57%	0.27
Arjmandi 2005 [24]	BMD TB (g/cm^2^)	SPI+	Non-soy AP	1 y	35	27	1.05	1.05	0.000%^2^	-5.03%, 5.03%	0.986
Kenny 2009 [23]	BMD TB (g/cm^2^)	SPI+	Milk+egg white	1 y	25	22	1.106	1.086	-0.17%	-1.06%, 072%	>0.05
		SPI-	Milk+egg white	1 y	24	22	1.129	1.086	0.20%	-.56%, 0.95%	>0.05
		SPI+	SPI-	1 y	25	24	1.106	1.129	-0.37%	-1.25%, 0.51%	>0.05
Vupadhyayula 2009 [27]	BMD TB (g/cm^2^)	SPI+	Milk	2 y	57	52	1.113	1.127	-0.29%	-1.03%, 0.45%	>0.05
		SPI-	Milk	2 y	48	52	1.114	1.127	0.08%	-0.82%, 0.66%	>0.05
		SPI+	SPI-	2 y	57	48	1.113	1.114	-0.21%	-2.83%, 2.41%	>0.05
Arjmandi 2005 [24]	BMC TB (g)	SPI+	Non-soy AP	1 y	35	27	2023	2022	0.40%^2^	-6.96%, 7.76%	0.944

^1^ AP, animal protein; BMC, bone mineral content; BMD, bone mineral density; CI, confidence interval; CP, comparator protein; FN, femoral neck; LS, lumbar spine; RCT, randomized controlled trial; SP, soy protein; SPI-, isoflavone-poor soy protein SPI+, isoflavone-rich soy protein; TB, total body; TH, total hip.

^2^ Imputation was used to calculate net change, r = 0.50

In the two three-arm RCTs [23, 27] comparing isoflavone-poor soy protein to animal protein and comparing isoflavone-rich to isoflavone-poor soy protein in 393 and 146 postmenopausal women, respectively, no significant differences in the net changes in LS BMD were found (Table 3).

### TH BMD

Two RCTs [24, 25] examined isoflavone-rich soy protein vs. animal protein’s effect on TH BMD in a total of 237 postmenopausal women. Both studies were one year in duration and administered protein via supplement powders to mix with a beverage in similar doses: 25 g protein/d [24] and 25.6 g protein/d [25]. Administered doses of isoflavones in the soy protein groups were 60 mg/d [24] and 97 mg/d [25]. The animal protein source was milk protein in one study [25] and an unspecified, non-soy protein in the second study [24]. Overall ROB was medium due to low compliance (<80%) and incomplete outcome data (i.e., dropout rate >20%) in one study [24] (S3 Table). Neither RCT comparing isoflavone-rich soy protein to animal protein intake found a significant difference in the net changes in TH BMD (Table 3).

### FN BMD

Three RCTs [23, 25, 27] examined isoflavone-rich soy protein vs. animal protein’s effect on FN BMD. Of these, two RCTs [23, 27] had a third, isoflavone-poor soy protein group, and thus also contributed data on the comparison between isoflavone-poor soy protein to animal protein as well as on the comparison between isoflavone-poor and isoflavone-rich soy protein. Two studies administered 25g protein/d via supplement powders to mix with a beverage and administered milk protein as the source of animal protein [25, 27], while a third study administered 18g protein/d via supplement powders to mix with a food or beverage and administered an egg white-milk protein mix as the source of animal protein [23]. Administered doses of isoflavones in the soy protein groups ranged from 35-97mg/d and study duration ranged from one to two years. Overall ROB was low, though it is important to note one study [23] did have incomplete outcome data (dropout rate >20% with no comparison between study completers and dropouts in per protocol analysis) (S3 Table).

**Fig 3:**
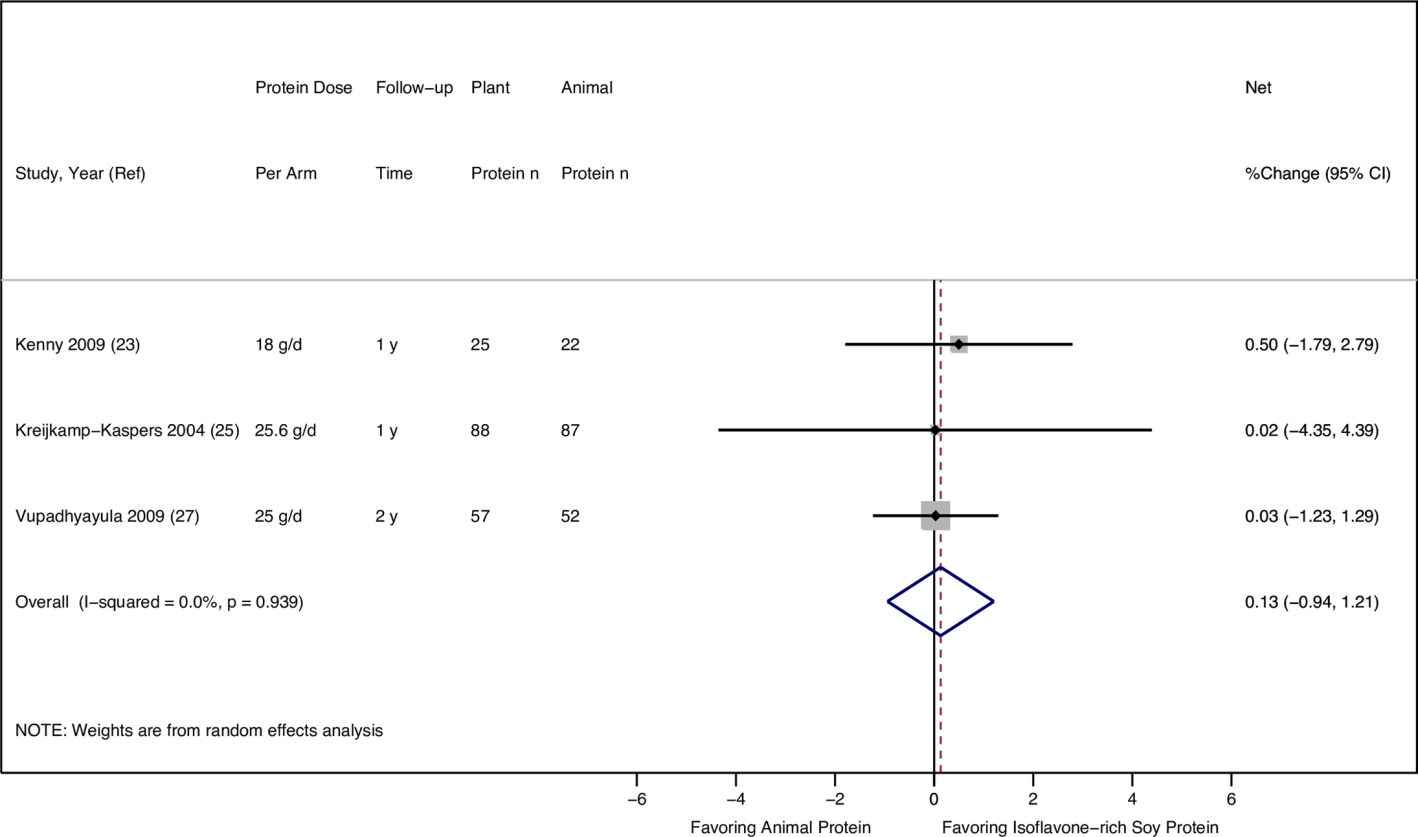
Effect of isoflavone-rich soy protein vs. animal protein intake on femoral neck BMD in postmenopausal women.

None of the three RCTs [23, 25, 27] comparing isoflavone-rich soy protein and animal protein intake found a significant difference in the net changes in FN BMD over one to two years (Table 3). The random-effects meta-analysis of the three RCTs in a total of 331 postmenopausal women also showed no difference in the effects of isoflavone-rich soy protein vs. animal protein intake on FN BMD, with no statistical heterogeneity (pooled mean percentage change: 0.13%, 95% CI: -0.94%, 1.21%, I^2^: 0.0%) (**Fig 3**).

In the two, three-arm RCTs [23, 27] comparing isoflavone-poor soy protein to animal protein and comparing isoflavone-rich to isoflavone-poor soy protein in 146 and 154 postmenopausal women, respectively, no significant differences in the net changes in FN BMD were found (Table 3).

### TB BMD

Three RCTs [23, 24, 27] examined isoflavone-rich soy protein vs. animal protein’s effect on TB BMD. Of these, two RCTs [23, 27] had a third, isoflavone-poor soy protein group, and thus also contributed data on the comparison between isoflavone-poor soy protein to animal protein as well as on the comparison between isoflavone-poor and isoflavone-rich soy protein. Protein dose and administration varied: two [24, 27] administered a supplement of 25 g protein/d to mix with a beverage and one study [23] administered 18g protein/d to mix with a food or beverage. Administered doses of isoflavones in the soy protein groups ranged from 35–90 mg/d, and animal protein sources varied across studies. Overall ROB was medium, due to low compliance in one study (<80%) [23] and incomplete outcome data in two studies (i.e., dropout rate >20%) [23, 24] (S3 Table).

**Fig 4:**
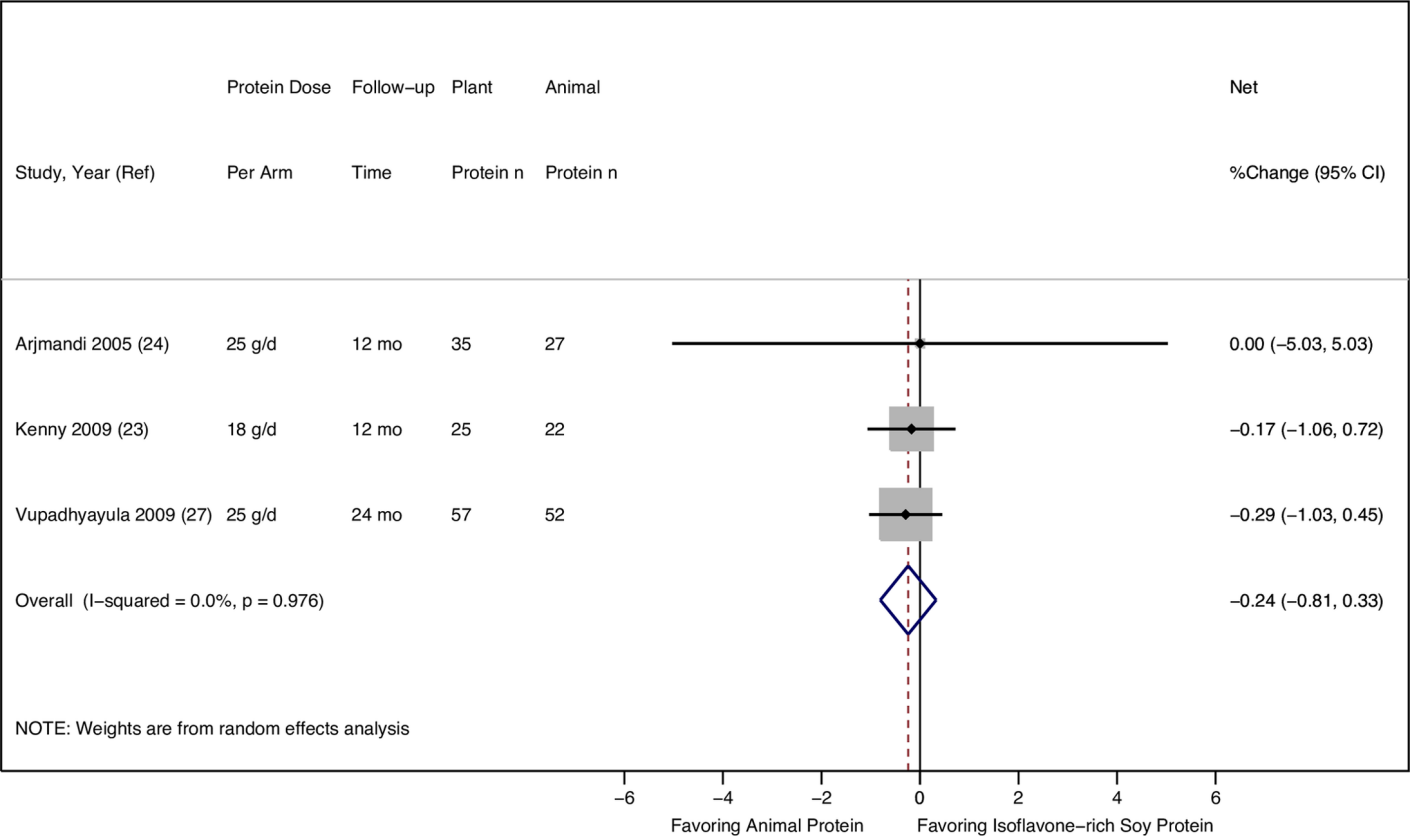
Effect of isoflavone-rich soy protein vs. animal protein intake on total body BMD in postmenopausal women.

None of the three RCTs comparing isoflavone-rich soy protein to animal protein found a significant difference in the net changes in TB BMD over one to two years (Table 3). The random-effects meta-analysis of the three RCTs in a total of 218 postmenopausal women also showed no difference in the effects of isoflavone-rich soy protein vs. animal protein intake on TB BMD, with no statistical heterogeneity (pooled mean percentage change: -0.24%, 95% CI: -0.81%, 0.33%, I^2^: 0.0%) (**Fig 4**).

In the two three-arm RCTs [23, 27] comparing isoflavone-poor soy protein to animal protein and comparing isoflavone-rich to isoflavone-poor soy protein in 146 and 154 postmenopausal women, respectively, no significant differences in the net changes in TB BMD were found (Table 3).

### TB BMC

Only one twelve-month RCT [24] examined isoflavone-rich soy protein vs. animal protein’s effect on TB BMC in 62 postmenopausal women. The study administered 25g soy (with 60 mg isoflavones/d) vs. unspecified, non-soy protein/d via both food and supplement powders to mix with a beverage, and did not find a significant difference in the net changes in TB BMC after one year (Table 3). However, the ROB for this RCT [24] was high, due to low compliance (<80%) and incomplete outcome data (i.e., dropout rate >20%) (S3 Table).

### BSAP

Five RCTs [19, 22–25] examined isoflavone-rich soy protein vs. animal protein’s effect on BSAP levels in 372 peri- and postmenopausal women. Of these, two RCTs [22, 23] had a third, isoflavone-poor soy protein group, and thus also contributed data on the comparison between isoflavone-poor soy protein to animal protein as well as on the comparison between isoflavone-poor and isoflavone-rich soy protein. Protein administration varied across studies: two studies [22, 25] administered a supplement powder to mix with a beverage, one administered a supplement powder to mix with a food or beverage[23], one administered both food and a supplement powder, and one administered a protein beverage [19]. Protein doses ranged from 18–40g/d. Isoflavones in the soy protein groups ranged from 35–97 mg/d and animal protein sources varied across studies. Overall ROB was medium, as two [19, 24] had low compliance (<80%) and two studies [23, 24] had incomplete outcome data (i.e., dropout rate >20%) (S3 Table).

There was no significant difference between isoflavone-rich soy protein and animal protein intake and changes in BSAP in one six-month [22] and three one-year RCTs [23–25] **(Table 4)**. One nine-month RCT [19] found those taking 25.6 g/d soy protein isolate plus 91.2 mg/d isoflavones had a significantly greater reduction in BSAP compared to those taking 25.6 g/d milk protein isolate (net change: 0.75 U/L, 95% CI 0.13, 1.37)).

**Table 4:** RCT biomarker results comparing isoflavone-rich soy protein, isoflavone-poor soy protein, and animal protein^1^.

Study Year [ref]	Outcome (units)	Soy Protein Group (SPI+/SPI-)	Comparator Protein Group (AP/ SPI-)	Study Length	SP n	CP n	SP Mean at BL	CP Mean at BL	Net change	95% CI	P-value
Alekel 2000 [22]	BSAP (μg/L)	SPI+	Whey	6 mo	24	21	NR	NR	No sig diff between groups (p>0.05, data in figure only)		
		SPI-	Whey	6 mo	24	21	NR	NR	No sig diff between groups (p>0.05, data in figure only)		
		SPI+	SPI-	6 mo	24	24	NR	NR	No sig diff between groups (p>0.05, data in figure only)		
Arjmandi 2005 [24]	BSAP (U/L)	SPI+	Non-soy AP	1 y	35	27	19.8	19.8	-0.40^2^	-3.59, 2.79	0.796
Evans 2007 [19]	BSAP (U/L)	SPI+	MPI	9 mo	21	22	SPI: 32.6 SPI+Ex: 25.6	MPI: 25.3 MPI+Ex: 28.5	0.75	0.13, 1.37	0.03
Kenny 2009 [23]	BSAP (U/L)	SPI+	Milk+egg white	1 y	25	22	24.7	26.8	0.60^2^	-4.61, 5.81	>0.05
		SPI-	Milk+egg white	1 y	24	22	21.2	26.8	-0.80	-5.22, 3.62	>0.05
		SPI+	SPI-	1 y	25	24	24.7	21.2	1.40	-2.68, 5.48	>0.05
Kreijkamp-Kaspers 2004 [25]	BSAP (μg/L)	SPI+	Milk	1 y	88	87	12.7	12.9	-0.23	-0.98, 0.53	0.55
Alekel 2000 [22]	NTx (nmol BCE/ mmol creatinine)	SPI+	Whey	6 mo	24	21	NR	NR	No sig diff between groups (p>0.05, data in figure only)		
		SPI-	Whey	6 mo	24	21	NR	NR	No sig diff between groups (p>0.05, data in figure only)		
		SPI+	SPI-	6 mo	24	24	NR	NR	No sig diff between groups (p>0.05, data in figure only)		
Kenny 2009 [23]	NTx (nmol BCE/ mmol creatinine)	SPI+	Milk+egg white	1 y	25	22	37.5	37.5	0.90^2^	-7.83, 9.63	>0.05
		SPI-	Milk+egg white	1 y	24	22	33.1	37.5	-0.40	-9.19, 8.39	>0.05
		SPI+	SPI-	1 y	25	24	37.5	33.1	1.30	-7.01, 9.61	>0.05
Murray 2003 [26]	NTx (nmol BCE)	SPI+	Whey+casein	6 mo	8	7	18.5	16.8	-1.60^2^	-6.91, 3.71	>0.05
Vupadhyayula 2009 [27]	NTx (nmol BCE)	SPI+	Milk	2 y	25	30	NR	NR	2.79	-1.98, 7.56	>0.05
		SPI-	Milk	2 y	22	30	NR	NR	4.13	-1.99, 10.25	>0.05
		SPI+	SPI+	2 y	25	22	NR	NR	-1.34	-5.72, 3.04	>0.05
Arjmandi 2005 [24]	DPD (nmol/mmol creatinine)	SPI+	Non-soy AP	1 y	35	27	5.2	5.3	0.16^2^	-0.35, 0.66	0.888
Evans 2007 [19]	CTX (ng/mL)	SPI+	MPI	9 mo	21	22	SPI: 0.61 SPI+Ex: 0.47	MPI: 0.49 MPI+Ex: 0.56	3.3	0.695, 5.905	0.02

^1^ BSAP, bone-specific alkaline phosphatase; CI, confidence interval; CTX, C-terminal telopeptide of type 1 collagen; diff, difference; DPD, deoxypyridinoline; MPI, milk protein isolate; NR, not reported; NTX, N-terminal telopeptide of type I collagen; RCT, randomized controlled trial; sig, significant; SPI+, isoflavone-rich soy protein isolate; SPI-, isoflavone-poor soy protein isolate.

^2^Imputation was used to calculate net change, r = 0.50

In the two, three-arm RCTs [22, 23] comparing isoflavone-poor soy protein to animal protein and comparing isoflavone-rich to isoflavone-poor soy protein in 91 and 94 women, respectively, no significant differences in the net changes in BSAP were found (Table 4).

### NTX

Four RCTs [22, 23, 26, 27] examined isoflavone-rich soy protein vs. animal protein’s effect on NTX levels in 162 peri- and postmenopausal women. Of these, three RCTs [22, 23, 27] had a third, isoflavone-poor soy protein group, and thus also contributed data on the comparison between isoflavone-poor soy protein to animal protein as well as on the comparison between isoflavone-poor and isoflavone-rich soy protein. Protein administration varied across studies: three studies [23, 26, 27] administered protein powder supplements and one study administered both protein powder and food [22]. The protein doses in each study ranged from 18–40 g/d and, in the soy groups, isoflavones ranged from 35–120 mg/d. Animal protein groups received milk protein sources in three studies [22, 26, 27] and an egg white-milk protein mix in one study [23]. Overall, ROB was medium, as two studies [23, 26] had incomplete outcome data (dropout rate >20%) (S3 Table).

None of the four RCTs comparing isoflavone-rich soy protein to animal protein found a significant difference in the net changes in NTX after six months to one year (Table 4). In the two, three-arm RCTs [22, 23] comparing isoflavone-poor soy protein to animal protein and comparing isoflavone-rich to isoflavone-poor soy protein in 133 and 144 women, respectively, no significant differences in the net changes in NTX were found (Table 4).

### C-terminal telopeptide of type 1 collagen (CTX)

One nine-month RCT [19] examined isoflavone-rich soy protein vs. animal protein’s effect on CTX levels in 43 postmenopausal women. The ROB for this study [19] was medium, due to low compliance (<80%) (S3 Table). The study administered a supplement powder containing 25.6g of soy to mix with a food or beverage vs. milk protein/d; the soy group also received a tablet containing 91.2 mg isoflavones/d while the milk protein group received a placebo tablet. The study found a significantly greater reduction in CTX in the isoflavone-rich soy group compared to the milk protein group (net change: 3.3 ng/mL, 95% CI 0.695, 5.905; Table 4).

### Deoxypyridinoline (DPD)

One twelve-month RCT [24] examined isoflavone-rich soy protein vs. animal protein’s effect on DPD levels in 62 postmenopausal women. The overall ROB for this study [24] was medium, due to low compliance (<80%) and incomplete outcome data (dropout rate >20%) (S3 Table). The study administered protein supplements to mix with a beverage containing 25 g of soy protein (with 60 mg isoflavones) vs. an unspecified, non-soy protein, and did not find a significant difference in the net change in DPD between groups (net change = 0.16 nmol/mmol creatinine, 95% CI -0.35, 0.66; Table 4).

### SOE Grading for RCTs

Evidence grading scores for soy isoflavone-poor protein, soy isoflavone-rich protein vs. animal protein intake by outcome are listed in **Tables 5 and 6.** No outcome received greater than a C-level or “limited” evidence grade due to the limited number of RCTs and lack of prospective cohort studies addressing each outcome. C-level evidence grade was assigned for LS, FN, and TB BMD and the bone turnover markers BSAP and NTX as only limited evidence suggests no significant difference between isoflavone-poor protein vs. animal protein. D-level evidence was assigned for CTX and DPD, as these outcomes were only examined by one study each. C-level evidence grade was assigned for LS, TH, FN, and TB BMD, TB BMC, and the bone turnover markers BSAP and NTX as only limited evidence suggests no significant difference between isoflavone-rich protein vs. animal protein. Several outcomes, including fractures of any type, falls, CTX, DPD, and certain BMD sites were not reported in any study and thus were assigned D-level or “inadequate” evidence grade. Several studies also had “unclear” or “high” ROB assessments, which also factored into the evidence grading (S3 Table).

**Table 5:** SOE grading: equal amounts of soy, isoflavone-rich protein vs. animal protein intake by outcome^1^.

Outcome	Studies (N, (Ref))		SOE Grade	Explanation
	RCTs	Cohort Studies		
BMD LS	4 [23–25, 27]	0	C	We conclude a C level of evidence that there is no significant difference between soy vs. animal protein intake on BMD loss in postmenopausal women. Four RCTs with medium ROB did not find a significant difference between groups.
BMD TH	2 [24, 25]	0	C	We conclude a C level of evidence that there is no significant difference between soy vs. animal protein intake on BMD loss in postmenopausal women. Two RCTs with medium ROB did not find a significant difference between groups.
BMD FN	3 [23, 25, 27]	0	C	We conclude a C level of evidence that there is no significant difference between soy vs. animal protein intake on BMD loss in postmenopausal women. Three RCTs with low ROB did not find a significant difference between groups.
BMD TB	3 [23, 24, 27]	0.	C	We conclude a C level of evidence that there is no significant difference between soy vs. animal protein intake on BMD loss in postmenopausal women. Three RCTs with medium ROB did not find a significant difference between groups.
BMC TB	1 [24]	0	D	We conclude a D level of evidence that there is no significant difference between soy vs. animal protein intake on TB BMC loss in postmenopausal women. One RCT with medium ROB did not find a significant difference between groups.
Falls	0	0	D	There is insufficient data to support a hypothesis: no study examined this association.
Fractures	0	0	D	There is insufficient data to support a hypothesis: no study examined this association.
BSAP	5 [19, 22–25]	0	C	We conclude a C level of evidence that there is no significant difference between soy vs. animal protein intake on BSAP in postmenopausal women. Four RCTs did not find a significant difference between groups. One six-month RCT found the soy group had a significantly greater reduction in BSAP compared to the milk protein group. ROB was medium overall.
CTX	1 [19]	0	D	We conclude a D level of evidence that soy protein causes a greater reduction in CTX compared to milk protein in postmenopausal women. Only one, 9-month RCT with medium ROB examined this association and found a greater reduction in the soy protein group vs. the milk protein group.
DPD	1 [24]	0	D	We conclude a D level of evidence that there is no significant difference between soy protein vs. animal protein intake on DPD in postmenopausal women. Only one, one-year RCT with medium ROB examined this association and did not find a significant difference.
NTX	4 [22, 23, 26, 27]	0	C	We conclude a C level of evidence that there is no significant difference between the effects of soy protein vs. animal protein intake on NTX in postmenopausal women. Four RCTs with medium ROB did not find a significant difference between groups.

^1^ BMD, bone mineral density; BMC, bone mineral content; BSAP, bone-specific alkaline phosphatase; CTX, C-terminal telopeptide of type 1 collagen; DPD, deoxypyridinoline; FN, femoral neck; LS, lumbar spine; NTX, N-terminal telopeptide of type 1 collagen; Ref, reference; TB, total body; TH, total hip; SOE, strength of evidence

**Table 6:** SOE grading: equal amounts of soy, isoflavone-poor protein vs. animal protein intake by outcome^1^.

Outcome	Studies (N, (Ref))		SOE Grade	Explanation
	RCTs	Cohort Studies		
BMD LS	2 [23, 27]	0	C	We conclude a C level of evidence that there is no significant difference between soy protein vs. animal protein intake on BMD loss in postmenopausal women. Two RCTs with low ROB comparing isoflavone-poor soy protein vs. animal protein did not find significant associations.
BMD TH	0	0	D	There is insufficient data to support a hypothesis: no study examined this association.
BMD FN	2 [23, 27]	0	C	We conclude a C level of evidence that there is no significant difference between soy protein vs. animal protein intake on BMD loss in postmenopausal women. Two RCTs with low ROB comparing isoflavone-poor soy protein vs. animal protein did not find significant associations.
BMD TB	2 [23, 27]	0.	C	We conclude a C level of evidence that there is no significant difference between soy protein vs. animal protein intake on BMD loss in postmenopausal women. Two RCTs with low ROB comparing isoflavone-poor soy protein vs. animal protein did not find significant associations.
BMC TB	0	0	D	There is insufficient data to support a hypothesis: no study examined this association.
Falls	0	0	D	There is insufficient data to support a hypothesis: no study examined this association.
Fractures	0	0	D	There is insufficient data to support a hypothesis: no study examined this association.
BSAP	2 [22, 23]	0	C	We conclude a C level of evidence that there is no significant difference between soy protein vs. animal protein intake on BSAP in postmenopausal women. Two RCTs with medium ROB comparing isoflavone-poor soy protein vs. animal protein did not find significant differences in the net changes in BSAP.
CTX	0	0	D	There is insufficient data to support a hypothesis: no study examined this association.
DPD	0	0	D	There is insufficient data to support a hypothesis: no study examined this association.
NTX	2 [22, 23]	0	C	We conclude a C level of evidence that there is no significant difference between soy protein vs. animal protein intake on NTX in postmenopausal women. Two RCTs with medium ROB comparing isoflavone-poor soy protein vs. animal protein did not find significant differences in the net changes in NTX.

^**1**^ BMD, bone mineral density; BMC, bone mineral content; BSAP, bone-specific alkaline phosphatase; CTX, C-terminal telopeptide of type 1 collagen; DPD, deoxypyridinoline; FN, femoral neck; LS, lumbar spine; NTX, N-terminal telopeptide of type 1 collagen; Ref, reference; TB, total body; TH, total hip; SOE, strength of evidence

### Findings from the prospective cohort studies

Given limited data from RCTs, we summarize key findings from the prospective cohort studies to supplement the findings from RCTs. Ten cohort studies [28–37] examining the associations between varying intakes of plant or animal protein and long-term bone health outcomes (follow-up ranged from 3 to 13 years, **S4 Table**). Protein intake was assessed in all studies using food frequency questionnaires (FFQs). Total plant and animal protein intake were included in statistical models either separately as categorical or continuous variables or together as a ratio (i.e., animal to plant protein intake ratio); and thus, none of these studies provide direct comparisons between plant and animal protein intake in relation to the bone health outcomes. Of the 10 cohort studies, five reported BMD outcomes, six reported fracture outcomes, and one reported fall outcome. The results are summarized in **S5 Table**.

Overall, most of cohort studies did not find statistically significant dose-response relationships between levels of plant or animal protein intake and bone loss (as measured by changes in BMD), and the associations between plant or animal protein intake and fracture outcomes were inconsistent across studies. Only one study [37] examined the associations between plant and animal protein intake and falls; the findings were not significant. Two cohort studies [28, 35] showed an interaction between calcium and sources of protein intake on the risk of fracture, with significant findings only among those with the lowest calcium intake (<417 mg/1000kcal [28] and 800 mg/d [35]). Among those with the lowest calcium intake, both studies found those in the highest animal protein quantile had significantly greater risk for fractures compared to the lowest animal protein quantile [28, 35]; one study [28] found those in the highest plant protein quantile had significantly lower risk of fracture compared to the lowest plant protein quantile, while the second study [35] reported no significant differences. Two studies examined the ratio of animal to plant protein intake; one found a higher ratio of animal to plant protein intake was associated with a higher risk of bone loss and hip fracture [36], while the second study found no association with hip fracture [35].

## Discussion and conclusions

Osteoporosis and low bone mass are a major health concern for an estimated 54 million Americans over the age of 50 years [38]. The relationship between dietary protein intake and the type of protein consumed has been a topic of great debate over the past several decades. This second systematic review, commissioned by the NOF, continues to further our understanding of the relationship between dietary protein intake to markers of bone health by assessing if significant differences in plant versus animal protein intakes exist.

Our systematic review found only C-level or “limited” evidence to suggest that there is no significant difference between isoflavone-rich, isoflavone-poor, or animal protein at several but not all BMD sites and the bone turnover markers NTX and BSAP. It is important to note that one of the four studies examining the outcome NTX by Murray et al. [26] did not find a significant difference in the net change of NTX between isoflavone-rich soy and animal protein groups. However, these negative findings are unsurprising considering the groups also received estradiol; taking HRT or estradiol would likely mask any effect of dietary protein on bone turnover and should be considered as a limiting factor on any dietary effect in future study designs. D-level or “inadequate” evidence was assigned for several BMD sites, falls, and fractures of any type, and thus no conclusions could be made regarding the effects of soy proteins vs. animal proteins on these outcomes. D-level was also assigned for the bone turnover markers CTX and DPD. One study [19] seemed to show an effect of higher SPI (91 mg/d) on attenuating the bone marker CTX in estrogen-deficient women compared to animal protein; this relationship should be considered as a direction for future studies.

Acid-producing diets are characterized by higher intakes of sulfur-containing amino acids from protein and cereal grains in relation to intake of fruits and vegetables [39, 40]. These amino acids are metabolized to sulfuric acid; an increased dietary acid load can cause low-grade metabolic acidosis and may lead to increased bone resorption and reduced bone mineral density [40]. In the Western world, the main contributors to the acid load from the diet are meat, fish, milk and dairy products, and eggs, followed by cereal grains [41]. Conversely, base-producing diets, characterized by relatively higher intake of fruits and vegetables, are rich in organic anion salts that are metabolized to alkaline salts such as bicarbonate [39, 42]. These metabolites can improve subclinical acidosis and may reduce bone turnover and preserve bone [40, 42]. However, the acid-base balance effect on bone hypothesis has been questioned on theoretical and experimental grounds because it was based on patients with chronic kidney disease and may not extrapolate to healthy adults [43]. This review is consistent with that interpretation of the literature. Though the included studies overall did not report urine and blood protein levels, calcium excretion rate, or patient metabolic or clearance rates, addressing them may be an interesting future research direction to better understand response variability.

The state-of-the-science does not support that consumption of soy protein is more advantageous on bone health outcomes as compared to animal proteins, or vice versa. All the included studies used isolated soy protein as the plant protein source. This choice may be due to the fact that soy protein, unlike most plant proteins, is a complete protein with high biological value and thus more similar to animal protein [44]. However, as the doses of soy and animal protein ranged from 18 to 40 g/d, some included studies may not have seen an effect due to the lower doses of protein prescribed. Future studies should consider high versus low protein doses and what protein dose may be needed to see an effect [45]. Future studies assessing intake of plant proteins aside from soy, such as from beans, legumes, quinoa, or pea or brown rice protein powders, as well as other animal proteins, such as from egg, are also needed to make generalizable dietary guidance regarding “plant” vs. “animal” protein. Additionally, the included studies focused on protein supplements; none of the included studies were total diet interventions or took into account protein purity or dietary composition. Further investigation of the effect of a variety of unrefined plant protein sources in the context of the food matrix compared to animal protein sources may be elucidating.

The effect of soy isoflavones on bone health has been recently reviewed [46]. Although consumption of soy products containing isoflavones have been associated with protection against hip fracture in Asia [47], several RCTs of isolated isoflavone preparations from soy in the US have shown almost no benefit to BMD [48–50]. The difference in findings between observational studies and RCTs has not been resolved, but differences in whole food vs. isolated compounds, populations being studied, and methodological approaches have all been considered.

None of the observational studies examined compared similar quantiles of plant and animal protein intake. Given the typical Western diet, it is not surprising that the quantiles of plant protein intake reported in all studies were lower than those for animal protein intake. The comparability of findings across observational studies was limited by these differing ranges within and between studies, as well as by the varying definitions of “plant” and “animal” protein in the studies’ FFQs when assessing usual protein intake. It is also important to note the potential for measurement error due to self-reported intake. Although these studies cannot directly answer the question of this systematic review, future observational studies examining the effects of sources of protein on bone health over long time periods can help generate future hypotheses for RCTs, particularly in instances where there is a paucity of intervention studies (e.g., the effect of different sources of protein intake on falls or fractures) and where intervention studies may be too costly.

Lastly, though the interaction between protein sources and calcium intake were not a focus of this systematic review, two of the cohort studies [28, 35] showed a significant interaction between protein intake and calcium for those in the lowest calcium quantile. In both studies, those in the highest animal protein quantile had significantly greater risk of fractures compared to those in the lowest animal protein quantile. Conversely, in one study [28] those in the highest plant quantile had a significantly lower risk of fracture compared to those in the lowest plant quantile, while the second study [35] reported no significant differences. Our previous systematic review [6] examined interactions between high versus low protein intake and calcium or vitamin D and found limited evidence did not support a synergistic effect of protein with calcium on LS BMD, TH BMD or forearm fractures; there was insufficient evidence for FN BMD and overall fracture outcomes. It thus may be of interest for future studies to take into account the sources of dietary protein in relation to calcium intake.

In conclusion, the results of this systematic review do not support that consumption of soy protein is more advantageous as compared to animal proteins, or vice versa. However, data for bone health outcomes were C-level or “limited” at best, for some but not all markers of bone health. There was insufficient evidence to draw conclusions regarding fractures and falls. We found no adverse effects of either soy or animal proteins on bone health. However, all interventions administered protein supplements, had limited study durations and sample sizes, and limited study populations to healthy, post-menopausal women. Larger, long-term RCTs and properly designed prospective cohort studies comparing dose-response relationships of soy and other plant proteins versus animal protein are greatly needed in the scientific literature.

## Supporting information

S1 File Analytical datasets. (XLSX)

S2 File PRIMA checklist. (DOC)

S1 Table Search strategy: Ovid MEDLINE®, 1946-October week 4 2016. The following search strategy was modified as needed for each database and conducted in: MEDLINE®, Scopus (including EMBASE from 1974), the Cochrane Central Register of Controlled Trials, Global Health, and Web-of-Science databases.
(DOCX)

S2 Table Sensitivity analysis^1^. ^1^BMD, bone mineral density; FN, femoral neck; CI, confidence interval; LS, lumbar spine.
(DOCX)

S3 Table RCT ROB assessments, by study (N = 7)^1^. ^1^H, high; L, low; NA, not applicable; RCT, randomized controlled trial; ROB, risk of bias; U, unclear.
(DOCX)

S4 Table Study characteristics summary table, cohort studies (N = 10)^1^. ^**1**^BMC, bone mineral content; BMD, bone mineral density; BMI, body mass index; CTX, C-terminal telopeptide of type 1 collagen; F%, percent female participants; FFQ, food frequency questionnaire; FN, femoral neck; gov, government; ind, industry; LS, lumbar spine; M, male; NA, not applicable; NP, non- profit; NR, not reported; ref, reference; SD, standard deviation; SE, standard error; TB, total body; TEI, total energy intake; TH, total hip; Vit D, vitamin D. ^2^ Animal and plant protein models adjusted for one another, while animal:plant protein ratio models adjusted for total protein intake.
(DOCX)

S5 Table Cohort studies’ results, by study (N = 10)^1^. ^1^BMC, bone mineral content; BMD, bone mineral density; BL, baseline; CI, confidence interval; E3N MGEN, Etude Epidémiologique de femmes de la Mutuelle Générale de l’Education Nationale; FN, femoral neck; HR, hazard ratio; IQR, interquartile range; IR, rate ratio; kcal, kilocalorie; LS, lumbar spine; ref, reference; MJ, megajoule; NR, not report; NHANES, National Health and Nutrition Examination Survey; NHS, Nurses’ Health Initiative; ref, reference; RR, relative risk; SD, standard deviation; SE, standard error; TB, total body; TEI, total energy intake; TH, total hip; Vit D, vitamin D; WHI, Women’s Health Initiative. ^2^ The association was examined in the supplemented group (calcium, Vit D) only; i.e., not examined in placebo group. ^3^ Mean follow-up reported only. ^4^ The number of women with incident fractures was reported and compared to fracture-free women. It is unclear if some women had more than one fracture or if that was measured. ^5^ Reported protein intake data are for N = 6510, not for the N = 4570 in the most adjusted model key findings reported here.
(DOCX)

## Abbreviations

BMC: bone mineral content
BMD: bone mineral density
CaR: calcium sensing receptor
CI: confidence interval
CTX: of type 1 collagen
DPD: deoxypyridinoline
FFQ: food frequency questionnaire
FN: femoral neck
LS: lumbar spine
NOF: National Osteoporosis Foundation
NTX: of type 1 collagen
PRISMA: Preferred Reporting Items for Systematic Reviews and Meta-analyses
RCT: randomized controlled trial
ROB: risk of bias
SD: standard deviation
SE: standard error
SOE: strength of evidence
TB: total body
TH: total hip
