# Supplementary material for: Animal versus plant protein and adult bone health: A systematic review and meta-analysis from the National Osteoporosis Foundation
Source: PLoS One. 2018 Feb 23;13(2):e0192459. doi: 10.1371/journal.pone.0192459 (PMC5825010; doi:10.1371/journal.pone.0192459)
Supplement: S1 Table — The following search strategy was modified as needed for each database and conducted in: MEDLINE®, Scopus (including EMBASE from 1974), the Cochrane Central Register of Controlled Trials, Global Health, and Web-of-Science databases. (DOCX) [file pone.0192459.s003.docx]

| 1 exp Dietary Proteins/  2 (diet$ adj2 protein$1).tw.  3 (protein adj1 (intake or consumption$1 or supplement$)).mp.  4 exp Soy Proteins/  5 exp Soybean Proteins/  6 (Soy$ protein$1).tw.  7 or/1-6 | Search terms for dietary protein consumption |
| --- | --- |
| 8 exp Bone Density/  9 exp Osteoporosis/  10 ((bone$1 or plate$1) adj3 mineral$).tw.  11 (bone adj2 (loss or turnover or densi$)).tw.  12 (Skelet$ adj2 (mineral$ or development$)).tw.  13 mineralization defect$1.tw.  14 Mineral$ content$1.tw.  15 BMC.tw.  16 Osteoporo$.tw.  17 Osteomalac$.tw.  18 exp Bone Development/  19 exp Osteogenesis/  20 fracture$1.tw.  21 exp Accidental Falls/  22 falls.tw.  23 exp "Bone and Bones"/  24 potential renal acid load.tw.  25 PRAL.tw.  26 net acid excretion.tw.  27 net endogenous acid production.tw.  28 or/8-27 | Search terms for bone health outcomes of interest |
| 29 7 and 28 | 29: Dietary protein intake AND bone health outcomes of interest |
| 30 limit 29 to systematic reviews  31 29 not 30  32 limit 31 to English language  33 32 not (animals not humans).mp.  34 limit 33 to (in vitro or lectures or news or "review")  35 33 not 34 | 30 and 31: Systematic reviews will be excluded  32 and 33: only studies in English and with humans will be included  34 and 35: in vitro studies, lectures, news and reviews will be excluded |
