# Supplementary material for: Animal versus plant protein and adult bone health: A systematic review and meta-analysis from the National Osteoporosis Foundation
Source: PLoS One. 2018 Feb 23;13(2):e0192459. doi: 10.1371/journal.pone.0192459 (PMC5825010; doi:10.1371/journal.pone.0192459)
Supplement: S2 Table — 1BMD, bone mineral density; FN, femoral neck; CI, confidence interval; LS, lumbar spine. (DOCX) [file pone.0192459.s004.docx]

| **Outcomes** | **r = 0.5** | **r = 0.2** | **r = 0.8** |
| --- | --- | --- | --- |
| LS BMD (%)，  Mean difference (95% CI) | 0.2403  (-0.7960, 1.2766) | 0.2410  (-0.8073, 1.2892) | 0.2378  (-0.7548, 1.2303) |
| FN BMD (%)，  Mean difference (95% CI) | 0.1322  (-0.9411, 1.2054) | 0.1347  (-0.9509, 1.2203) | 0.1230  (-0.9048, 1.1508) |
| TB BMD (%)，  Mean difference (95% CI) | -0.24  (-0.8059, 0.3282) | -0.2400  (-0.8084, 0.3284) | -0.2344  (-0.7961, 0.3273) |
