# Supplementary material for: Animal versus plant protein and adult bone health: A systematic review and meta-analysis from the National Osteoporosis Foundation
Source: PLoS One. 2018 Feb 23;13(2):e0192459. doi: 10.1371/journal.pone.0192459 (PMC5825010; doi:10.1371/journal.pone.0192459)
Supplement: S3 Table — 1H, high; L, low; NA, not applicable; RCT, randomized controlled trial; ROB, risk of bias; U, unclear. (DOCX) [file pone.0192459.s005.docx]

| **Author, Year (Ref)** | **Sequence generation** | **Allocation sequence concealment** | **Blinding of participants** | **Blinding of personnel** | **Blinding of outcome assessors** | **Compliance** | **Groups comparable at baseline** | **Incomplete outcome**  **data** | **Selective outcome reporting** |
| --- | --- | --- | --- | --- | --- | --- | --- | --- | --- |
| Alekel 2000 [22] | U | U | L | L | L | L | L | L | L |
| Arjmandi 2005 [24] | U | U | L | L | L | H | L | H | L |
| Evans 2007 [19] | L | L | L | L | L | H | L | L | L |
| Kenny 2009 [23] | U | U | L | L | L | L | L | H | L |
| Kreijkamp-Kaspers 2004 [25] | L | L | L | L | L | L | L | L | L |
| Murray 2003 [26] | L | U | L | L | L | L | L | H | L |
| Vupadhyayula 2009 [27] | L | L | L | L | L | L | L | L | L |
