# Supplementary material for: Animal versus plant protein and adult bone health: A systematic review and meta-analysis from the National Osteoporosis Foundation
Source: PLoS One. 2018 Feb 23;13(2):e0192459. doi: 10.1371/journal.pone.0192459 (PMC5825010; doi:10.1371/journal.pone.0192459)
Supplement: S4 Table — 1BMC, bone mineral content; BMD, bone mineral density; BMI, body mass index; CTX, C-terminal telopeptide of type 1 collagen; F%, percent female participants; FFQ, food frequency questionnaire; FN, femoral neck; gov, government; ind, industry; LS, lumbar spine; M, male; NA, not applicable; NP, non- profit; NR, not reported; ref, reference; SD, standard deviation; SE, standard error; TB, total body; TEI, total energy intake; TH, total hip; Vit D, vitamin D. 2 Animal and plant protein models adjusted for one another, while animal:plant protein ratio models adjusted for total protein intake. (DOCX) [file pone.0192459.s006.docx]

| **First author, pub year [ref] (country), cohort** | **Participants** | **Total enrolled/ analyzed (N) [F%]** | **Baseline mean age (SD), age range** | **BMI (kg/m^2^) or Body weight (kg), mean (SD)** | **Dietary protein assessment methods** | **Confounders adjusted for** | | **Study Length (y)** | **Outcomes assessed (Endpoint (1°, 2° or NR))** | **Funding source** |
| --- | --- | --- | --- | --- | --- | --- | --- | --- | --- | --- |
| Dargent-Molina, 2008 [28] (France) E3N (Etude Epidémiologique de femmes de la Mutuelle Générale de l’Education Nationale [MGEN]) | Post-menopausal women | 40,224/ 36,217 [100] | Crude NR, 40-65  No Fracture:  56.1 (5.5) Fracture: 57.1 (5.6) | BMI: Crude NR  No Fracture: 23.2 (3.3) Fracture: 23.3 (3.4) | Dietary questionnaire with FFQ | Total nonalcoholic energy intake, calcium, age, BMI, parity, maternal history of hip fracture, ever use of postmenopausal hormonal therapy use, physical activity, smoking, alcohol | | 12 | All fracture (NR) | combo (gov, non-p and ind) |
| Dawson-Hughes, 2002 [29] (USA)  NA | Healthy older adults | 389/342  [53] | Crude NR  By protein (% total kcal): T1 (9.64 -15.49) Supplement:70 (5) Placebo: 71 (5) T2 (15.53 -18.15) Supplement:71 (4) Placebo: 71 (5) T3 (18.16-29.14) Supplement:70 (4) Placebo: 71 (5) | Weight: Crude NR By protein (% total kcal):  T1:  Supplement: 75 (14)  Placebo: 75 (14) T2:  Supplement: 74 (14)  Placebo: 76 (15) T3:  Supplement: 76 (13)  Placebo: 72 (14) | 126-item Willett FFQ | Sex, age, weight, TEI, dietary calcium, physical activity score, smoking | | 3 | BMD (FN, TB) (NR) | gov |
| Feskanich, 1996 [30] (USA)  Nurses' Health Study (NHS) | Middle-aged women | 98,462/ 85,900 [100] | Crude NR, 30-55   By total protein (g/d):  <68: 45.7 (7.2) 68-77: 46.1 (7.1) 78-85: 46.4 (7.1) 86-95: 46.8 (7.2) >95: 47.5 (7.1) | BMI: Crude NR  By total protein (g/d): <68: 24.0 (4.4)  68-77: 24.1 (4.3)  78-85: 24.3 (4.4)  86-95: 24.5 (4.4)  >95: 25.2 (4.6) | Semi-quantitative FFQ | Age, BMI, postmenopausal hormone use and status, thyroid hormone medication, thiazide diuretics, rigorous physical activity, smoking, alcohol, caffeine, questionnaire time period | | 12 | Fracture (hip, forearm) (NR) | gov |
| Hannan, 2000 [31] (USA) Framingham Osteoporosis Study | Elderly adults | 855/615 [64] | 75 (4.4), 68-91 | Weight: 70.6 (14.7) | 126-item Willett FFQ | | TEI, calcium, age, sex, weight, weight change, height, current estrogen use, smoking, alcohol, physical activity | 4 | BMD (FN, LS) (NR) | gov |
| Langsetmo, 2015 [32] (Canada) Canadian Multicentre Osteoporosis Study | Adults | 6,510/ 4,661 [71] | Crude NR, 50+ | Crude NR | FFQ | | Age, BMI, height, TEI, study center, education, smoking, alcohol intake, physical activity, sedentary hours, calcium and Vit D supplement use; in women only: hormone therapy, bisphosphonate use, diagnosis of osteoporosis | 13 | BMD (TH, LS), All fracture (NR) | Combo (gov, ind) |
| Munger, 1999 [33] (USA)  Iowa Women's Health Study | Post-menopausal women | 41,837/ 32,050 [100] | Crude NR, 55-69  No fracture:  61.4 (4.2)  Fracture: 63.2 (4.7) | BMI: Crude NR  No fracture:  27.0 (5.1)  Fracture: 24.4 (3.7) | FFQ | | Age, BMI, parity, smoking, alcohol, physical activity | 3 | Hip Fracture (NR) | combo  (non-p, gov) |
| Promislow, 2002 [34] (USA)  Rancho Bernardo Heart and Chronic Disease Study | Adults-men and women 55+y | 1,526/ 960  [58] | Crude NR, 55-92  F: 71.2 (8.7) M: 70.0 (8.5) | BMI: Crude NR  F: 24.6 (3.7) M: 26.4 (3.4) | 128-item Harvard-Willett FFQ | | TEI, total calcium, age, BMI, change in body weight, diabetes status, number years post-menopausal, thiazides, thyroid hormones, steroids, estrogen, physical activity, smoking, alcohol | 4 | BMD (TH, FN, LS) (NR) | gov |
| Sahni, 2010 [35] (USA) Framingham Offspring Study | Adults-men and women | 3,656/ 3,656  [53] | Crude: 55 (NR)  M: 55.3 (9.9)  F: 54.9 (9.8) | BMI: Crude NR M: 28.1 (4.1) F: 26.8 (5.5) | Semi-quantitative 126-item Willett FFQ | | TEI, dietary calcium, calcium supplement use, Vit D, sex, age, weight, height, menopause status, physical activity index, smoking status; animal, plant or total protein intake^2^ | 12 | Hip Fracture (NR) | gov |
| Sellmeyer 2001 [36] (USA), Study of Osteoporotic Fractures | Post-menopausal women | 1061/1035 [100] | Crude: NR, >65y Low ratio: 74.3 (5.4)  Medium ratio: 73.2 (4.9)  High ratio: 72.5 (4.5) | BMI: Crude: NR Low ratio: 25.6 (4.6)  Medium ratio: 26.5 (4.7)  High ratio: 26.7 (4.9) | 63-Item FFQ | | TEI, total calcium intake (dietary+supplements), energy-adjusted total protein intake, age, weight, current estrogen use, physical activity, smoking status, alcohol intake | Mean (SD): 7.0 (1.5) | FN BMD, Hip Fracture (NR) | gov |
| Zoltick, 2011 [37] (USA) Framingham Original Cohort Study | Older adults- men and women | 807/807  [63] | 75 (4.8), 67-93 | BMI: 26.7 (4.6) | Semi-quantitative 126-item Willett FFQ | | TEI, dietary calcium, calcium supplement use, dietary Vit D, Vit D supplement use, age, sex, weight, height, falls in past year, physical activity, alcohol, smoking | 2 | Falls (NR) | gov |
