# Supplementary material for: Animal versus plant protein and adult bone health: A systematic review and meta-analysis from the National Osteoporosis Foundation
Source: PLoS One. 2018 Feb 23;13(2):e0192459. doi: 10.1371/journal.pone.0192459 (PMC5825010; doi:10.1371/journal.pone.0192459)
Supplement: S5 Table — 1BMC, bone mineral content; BMD, bone mineral density; BL, baseline; CI, confidence interval; E3N MGEN, Etude Epidémiologique de femmes de la Mutuelle Générale de l’Education Nationale; FN, femoral neck; HR, hazard ratio; IQR, interquartile range; IR, rate ratio; kcal, kilocalorie; LS, lumbar spine; ref, reference; MJ, megajoule; NR, not report; NHANES, National Health and Nutrition Examination Survey; NHS, Nurses’ Health Initiative; ref, reference; RR, relative risk; SD, standard deviation; SE, standard error; TB, total body; TEI, total energy intake; TH, total hip; Vit D, vitamin D; WHI, Women’s Health Initiative. 2 The association was examined in the supplemented group (calcium, Vit D) only; i.e., not examined in placebo group. 3 Mean follow-up reported only. 4 The number of women with incident fractures was reported and compared to fracture-free women. It is unclear if some women had more than one fracture or if that was measured. 5 Reported protein intake data are for N = 6510, not for the N = 4570 in the most adjusted model key findings reported here. (DOCX) [file pone.0192459.s007.docx]

| **Study Year (ref), cohort** | **Population** | **Protein intake  (mean (SD) at BL, categories)** | **Study Length** | **Outcome** | **Total N Analyzed** | **Number of events** | **Key Findings** |
| --- | --- | --- | --- | --- | --- | --- | --- |
| Dargent-Molina 2008 (28), E3N MGEN | Post-menopausal women | Animal protein (g/1000kcal):  Q1: <22.42  Q2: 22.42-27.75  Q3: 27.75-33.52  Q4: >33.52  Plant protein (g/1000kcal):  Q1: <10.07  Q2: 10.07-12.01  Q3: 12.01-14.12  Q4: >14.12  Calcium (mg/1000kcal)  Q1: <417  Q2: 417.3–500.6  Q3: 500.6–604.3  Q4: >604.3  Significant calcium*protein interaction | 12 y | Fractures- Overall | 36,217 | 2,408^4^ | Calcium*protein interaction:  Animal protein Q1+Calcium Q1: ref  Animal protein Q4 + Calcium Q1: RR 1.66,  95% CI 1.29-2.13  Other results NR  p for trend <0.0001  Plant protein Q1+Calcium Q1: ref  Plant protein Q4 + Calcium Q1: RR 0.68,  95% CI 0.53-0.87  Other results NR  p for trend <0.0001 |
| Dawson-Hughes 2002 (29) NA | Healthy older adults 65+ | Mean total protein (g): 79.1 (25.6)  % total kcal animal protein intake, stratified by total protein intake tertiles (% total kcal)^2^  T1 9.64-15.49: 8.8(1.7)  T2 15.53-18.15: 11.3 (1.5)  T3 18.16-29.14: 14.9 (2.1)  Sig effect by total protein tertile,  p<0.05  % total kcal plant protein intake, stratified by total protein intake tertiles (% total kcal)^2^  T1 9.64-15.49: 5.0 (1.1)  T2 15.53-18.15: 5.5 (1.2)  T3 18.16-29.14: 5.2 (1.0)  No sig diff in plant protein intake by  total protein tertile, p>0.05 | 3 y | BMD FN | 342 | NA | In the supplemented group (calcium, Vit D), animal protein as a percent of total protein was not significantly associated with change in BMD (p>0.05, data not shown) |
|  |  |  |  | BMD TB | 342 | NA | In the supplemented group (calcium, Vit D), animal protein as a percent of total protein was not significantly associated with change in BMD (p>0.05, data not shown) |
| Feskanich 1996 (30), NHS | Middle-aged women | Animal protein (g/d)  Q1: <51  Q2: 52-61  Q3: 62-69  Q4: 70-80  Q5: >80  Plant protein (g/d)  Q1: <12  Q2: 12-14  Q3: 15-16  Q4: 17-19  Q5: >19 | 12 y | Fractures- Hip | 85,900 | 234 | Animal protein  Q1: ref  Q2: RR 1.07, 95% CI 0.73-1.59  Q3: RR 0.95, 95% CI 0.63-1.42  Q4: RR 0.92, 95% CI 0.61-1.38  Q5: RR 0.98, 95% CI 0.65-1.47  p for trend=0.70  Plant protein  Q1: ref  Q2: RR 0.98, 95% CI 0.64-1.49  Q3: RR 0.92, 95% CI 0.60-1.41  Q4: RR 0.94, 95% CI 0.62-1.43  Q5: RR 1.11, 95% CI 0.75-1.66  p for trend=0.58 |
| Feskanich 1996 (30), (cont’d) |  |  |  | Fractures- Forearm | 85,900 | 1628 | Animal protein  Q1: ref  Q2: RR 1.15, 95% CI 0.98-1.35  Q3: RR 1.11, 95% CI 0.94-1.30  Q4: RR 1.25, 95% CI 1.07-1.46  Q5: RR 1.25, 95% CI 1.07-1.46  p for trend=0.004  Plant protein  Q1: ref  Q2: RR 1.01, 95% CI 0.87-1.18  Q3: RR 1.08, 95% CI 0.93-1.26  Q4: RR 1.01, 95% CI 0.86-1.18  Q5: RR 0.90, 95% CI 0.77-1.06  p for trend=0.17 |
| Hannan 2000 (31), Framingham Osteoporosis Study | Elderly adults | Mean animal protein intake (g): 45.7 (19.1)  Animal protein intake by quartile (g/d):  Q1: 4-32  Q2: 33-43  Q3: 44-57  Q4: 58-132  Animal protein intake by quartile (% total kcal)  Mean %: 10.6 (3.5)  Q1: 1.9-8.2  Q2: 8.3- 10.3  Q3: 10.4-12.5  Q4: 12.6-23.4  Plant protein intake: Mean and by quartile NR | 4 y | BMD FN | 615 | NA | Beta**:** Lower % energy from animal protein was significantly associated with greater BMD loss (p<0.03, data not shown); plant protein was not a significant predictor (p>0.05, data not shown)  Animal protein (in quartiles, % total kcal)-  Q1 LSM -3.95, SE 0.69, p< 0.05  Q2 LSM -3.83, SE 0.72, p<0.05  Q3 LSM -3.02, SE 0.73, p>0.05  Q4 LSM -2.15, SE 0.73, ref  Bone loss did not significantly differ by plant protein quartiles (all p>0.05, data in figure only). |
|  |  |  |  | BMD LS | 615 | NA | Beta: Lower % energy from animal protein was significantly associated with greater BMD loss (p<0.02, data not shown); non-animal protein was not a significant predictor of bone loss (p>0.05, data not shown)  Animal protein:  Q1: LSM -3.79, SE 0.99, 0.05<p<0.10  Q2: LSM -2.55, SE 1.0, p>0.05  Q3: LSM -1.24, SE 1.0, p>0.05  Q4: LSM -1.65, SE 1.1, ref  Bone loss did not significantly differ by non-animal protein quartiles (all p>0.05, data not shown). |
| Langsetmo 2015 (32), Canadian Multicentre Osteoporosis Study | Adults 50+y  (All men 50+y and restricted to post-menopausal women 50+y) | Protein intake (g), Median(IQR)^5^:  Dairy protein: 11.6 (6.5-21.7)  Non-dairy animal protein: 17.6 (12.8-  23.0)  Plant-based protein: 24.3 (18.8-31.0)  Protein intake (%TEI), Median(IQR)^5^:  Dairy protein intake: 3.0 (1.8-4.8)  Non- dairy protein intake: 4.4 (3.3-  5.6)  Plant protein intake: 6.1 (5.3-7.0) | 13 y | BMD TH | 6510 | NA | 5-year BMD change (Beta, 95% CI, g/cm^2^):  Dairy protein (%TEI):  Men 25-49 y: 0.001 (-0.003, 0.005)  Premenopausal women 25-49: 0.002 (-0.001, 0.005)  Men 50+: 0.001 (-0.002, 0.003)  Postmenopausal women 50+: 0.002 (0.000, 0.003), P<0.05  Non-dairy protein (%TEI):  Men 25-49 y: 0.000 (-0.004, 0.004)  Premenopausal women 25-49: 0.001 (-0.002, 0.005)  Men 50+: 0.000 (-0.001, 0.004)  Postmenopausal Women 50+: 0.000 (-0.002, 0.002)  Plant protein (%TEI):  Men 25-49 y: 0.001 (-0.003, 0.005)  Premenopausal women 25-49: -0.003 (-0.006, 0.001)  Men 50+: 0.001 (-0.002, 0.003)  Postmenopausal women 50+: 0.000 (-0.002, 0.002) |
|  |  |  |  | BMD LS | 6510 | NA | 5-year BMD change (Beta, 95% CI, g/cm^2^):  Dairy protein (%TEI):  Men 25-49 y: -0.002 (-0.008, 0.004)  Premenopausal Women 25-49: 0.001 (-0.003, 0.005)  Men 50+: 0.002 (-0.002, 0.005)  Postmenopausal Women 50+: 0.001 (-0.001, 0.003)  Non-dairy protein (%TEI):  Men 25-49 y: -0.001 (-0.006, 0.005)  Premenopausal Women 25-49: -0.001 (-0.005, 0.003)  Men 50+: 0.000 (-0.003, 0.004)  Postmenopausal Women 50+: 0.001 (-0.001, 0.003)  Plant protein (%TEI):  Men 25-49 y: 0.000 (-0.005, 0.005)  Premenopausal Women 25-49: -0.002 (-0.006, 0.003)  Men 50+: 0.001 (-0.002, 0.005)  Postmenopausal Women 50+: -0.003 (-0.005, 0.000), p<0.05 |
|  |  |  |  | Fractures-Overall | 4570 | 518 | There was no significant difference between animal vs. plant protein intake and overall fractures (p>0.05, data nor reported) |
| Munger 1999 (33), Iowa Women's Health Study | Post-menopausal women | Quartiles animal protein intake (g/MJ)  Q1: <6.48  Q2: 6.48–7.82  Q3: 7.82–9.26  Q4: >9.26  Quartiles vegetable protein intake (g/MJ)  Q1: <2.51  Q2: 2.51–2.88  Q3: 2.88–3.28  Q4: >3.28 | 104,338 person-y | Fractures- Hip | 32,050 | 44 | Animal protein intake:  Q1: ref  Q2: RR 0.59, 95% CI 0.26, 1.34  Q3: RR 0.63, 95% CI 0.28, 1.42  Q4: RR 0.31, 95% CI 0.10, 0.93  P for trend: 0.037  Plant protein intake:  Q1: Ref  Q2: RR 1.15, 95% CI 0.38, 3.42  Q3: RR 1.86, 95% CI 0.69, 4.98  Q4: RR 1.92, 95% CI 0.72, 5.11  P for trend: 0.11 |
| Promislow 2002 (34), Rancho Bernardo Heart and Chronic Disease Study | Adults- men and women 55+ | Mean animal protein intake (g)  Men: 50.7 (18.8)  Women: 48.5 (19.4)  Mean vegetable protein intake (g)  Men: 23.1 (8.0)  Women: 22.7 (9.3) | 4 y | BMD LS | 954 | NA | There was no significant association between animal or plant protein intake and rate of bone loss in either men or women (p>0.1, data not shown). |
|  |  |  |  | BMD FN | 960 | NA | There was no significant association between animal or plant protein intake and rate of bone loss in either men or women (p>0.1, data not shown). |
|  |  |  |  | BMD TH | 954 | NA | There was no significant association between animal or plant protein intake and rate of bone loss in either men or women (p>0.1, data not shown). |
| Sahni 2010 (35), Framingham Offspring Study | Adults- men and women | Mean animal protein intake (g)  Men: 54.3(22)  Women: 52.5(22)  Mean plant protein intake (g)  Men: 24.6(9)  Women: 23.1(9)  Mean animal/plant protein ratio:  Men: 2.4(1)  Women: 2.4(1)  Tertiles total protein intake: NR | 12 y | Fractures- Hip | 3,656 | Women: 34 Men: 10 | (Sig protein*calcium interaction)  < 800 mg calcium/d by tertiles of total protein intake:  Animal protein intake:  T1: ref  T2: HR 0.97, 95% CI 0.33-2.78  T3: HR 3.17, 95% CI 1.30–7.78  p for trend= 0.01  Plant protein intake:  T1: ref  T2: HR 1.10, 95% CI 0.46-2.64  T3: HR 0.60, 95% CI 0.20–1.85  p for trend= 0.34  ≥800 mg calcium/d, by tertiles of total protein intake:   Animal protein intake:  T1: ref  T2: HR 1.51, 95% CI 0.43-5.31  T3: HR 0.32, 95% CI 0.05-2.08  p for trend= 0.33  Plant protein intake:   T1: ref  T2: HR 0.59, 95% CI 0.17-2.04  T3: HR 0.23, 95% CI 0.05-1.03  p for trend= 0.06  Animal/plant protein ratio: There was no significant association with risk of hip fracture. |
| Sellmeyer 2001 (36), Study of Osteoporotic Fractures | Post-menopausal women | By ratios of animal/vegetable protein intake:  Animal/vegetable protein intake ratio:  Low: 1.2 (0.27)  Medium: 2.3 (0.44)  High: 4.2 (1.2)  Animal protein intake (g):  Low: 23.6(6.8)  Medium: 35.1(6.5)  High:48.2 (9.3)  Vegetable protein intake (g):  Low: 19.6(3.6)  Medium: 15.4 (2.7)  High:11.6 (2.8)  P for trend for all above categories: 0.001 | 7 y^3^ | BMD FN | 742 | NA | BMD loss was significantly higher in women with a high ratio of animal:vegetable protein intake (0.78%/y) compared to those with a low ratio of intake (0.21%/y, p=0.02). There was no significant difference between those with a medium and low ratio of intake (data in figure only, p>0.05). |
|  |  |  |  | Fractures- Hip | 1035 | 48 | Ratios of animal/vegetable protein intake:  Low ratio: ref  Medium ratio: 2.1, p>0.05  High ratio: RR 3.7, p=0.04 |
| Zoltick 2011 (37), Framingham Original Cohort Study | Older adults- men and women | Mean animal protein intake (g): 46 (19.1)  Mean plant protein intake (g) 23 (8.8)  Animal and plant protein intake by tertile: data reported in figure only | 2 y | Falls | 772 | Rate falls/y: 0.45 (Range 0-15 falls) 1 fall- 27%  2 falls- 8% | IR per 1 SD difference in:  Animal protein intake: 0.96, 95% CI 0.79, 1.16  Plant protein intake: 0.88, 95% CI 0.68, 1.14 IR by tertile animal protein intake:  T1: ref  T2: IR 0.80, 95% CI 0.55–1.16  T3: IR 0.88, 95% CI 0.60–1.27 |
